# Primary Carnitine Deficiency: A Rare, Reversible Metabolic Cardiomyopathy

**DOI:** 10.1155/2018/3232105

**Published:** 2018-09-13

**Authors:** Stephen Tomlinson, John Atherton, Sandhir Prasad

**Affiliations:** Department of Cardiology, Royal Brisbane and Women's Hospital, Cnr Butterfield St and Bowen Bridge Road, Herston, QLD 4029, Australia

## Abstract

A 24-year-old female with a diagnosis of primary carnitine deficiency, a rare inherited metabolic disorder predominantly described in the paediatric literature that causes cardiomyopathy, presented for evaluation after three months of nonadherence with prescribed carnitine therapy. Initial echocardiography demonstrated severe left ventricular dilation (104 ml/m^2^) (normal < 76 ml/m^2^) with moderate systolic dysfunction (ejection fraction 40%) and severe right ventricular dilation with mild systolic dysfunction. Carnitine replacement was commenced, and a cardiac magnetic resonance imaging (MRI) performed five days later demonstrated dramatic improvement in biventricular function with normalization of left and right ventricular systolic function. To our knowledge, this is only the second case describing the rapid reversal of cardiomyopathy in an adult patient with this rare condition.

## 1. Case

**Figure 1:**
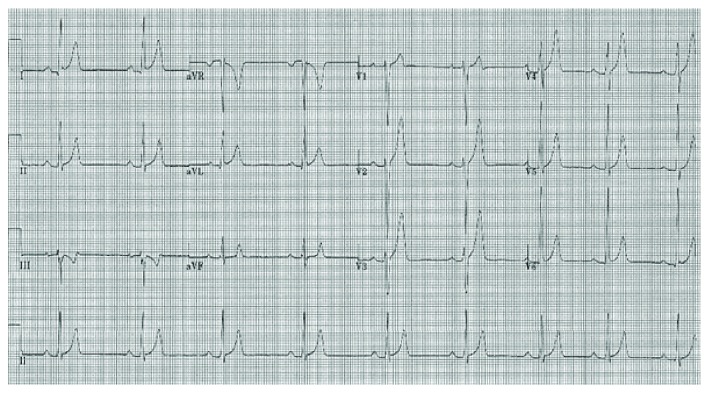
12-lead electrocardiogram taken on admission demonstrating peaked T waves and short QT interval.

A 24-year-old female presented to the Emergency Department of a tertiary hospital in Brisbane with a 48-hour history of diarrhoea and vomiting. Her medical history included primary carnitine deficiency, a rare inherited metabolic disorder that causes cardiomyopathy, which was diagnosed as asymptomatic during her infancy following the death of her infant brother from cardiomyopathy related to the condition. Her diagnosis was established by impaired cultured fibroblast carnitine uptake. Her genotype is unknown. She was prescribed oral carnitine replacement, 400 mg TDS, but had been not compliant with this therapy for the preceding three months. She reported no history suggestive of cardiac failure or arrhythmia. Cardiovascular and respiratory examination was normal. Chest X-ray revealed an increased cardiothoracic ratio. Electrocardiogram demonstrated enlarged peaked T waves and a short QT interval (Figure 1). Her serum total carnitine level was 4 *μ*mol/l (21–70). Intravenous carnitine replacement was commenced, and she was admitted for telemetry and cardiac evaluation. Her gastrointestinal symptoms resolved early in her admission and did not reoccur.

**Figure 2:**
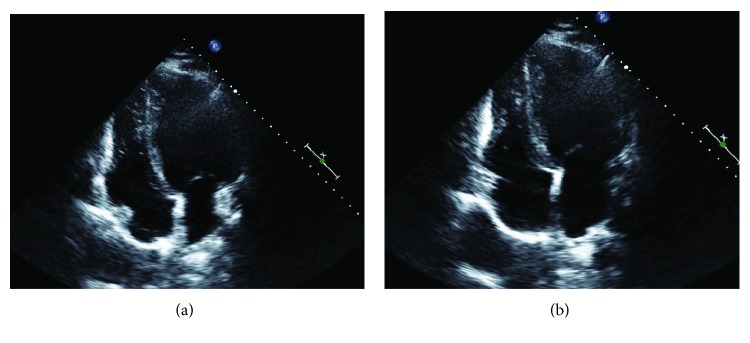
Echocardiogram on the day of presentation in (a) diastole and (b) systole (bar scale: one integer = 1 cm).

Echocardiography performed within 24 hours of commencing carnitine replacement revealed a dilated cardiomyopathy. The left ventricle was severely dilated with a left ventricular end diastolic volume index of 104 ml/m^2^ (normal < 76 ml/m^2^) with mild concentric wall thickening with a maximum wall thickness of 16 mm. The ejection fraction was 40% by Simpson's method with global hypokinesis. Grade II diastolic dysfunction was present. The right ventricle was severely dilated with mild systolic dysfunction (Figure 2).

The patient was transitioned from IV to PO carnitine, 400 mg TDS, and was commenced on bisoprolol 2.5 mg mane and perindopril 2.5 mg mane. Plasma carnitine concentration normalized within 24 hours and was sustained within normal limits for the duration of the admission on serial testing. Continuous telemetry monitoring demonstrated a single run of polymorphic ventricular tachycardia with a rate of 150 bpm. A CT coronary angiogram revealed no coronary atheroma. Screening for other causes of cardiomyopathy was not undertaken given the known carnitine deficiency.

**Figure 3:**
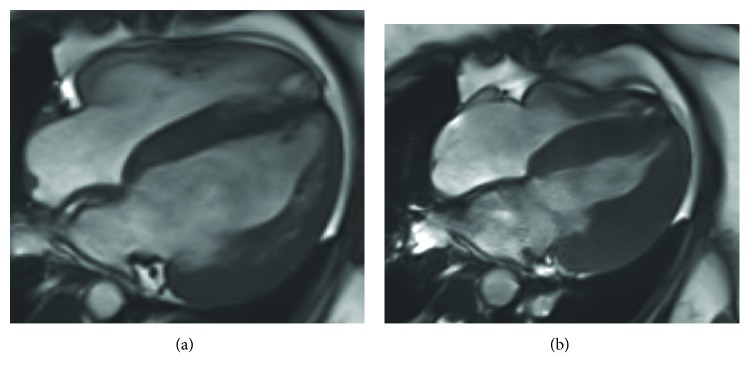
Cardiac MRI postcarnitine replacement in (a) diastole and (b) systole.

A cardiac MRI performed five days after the initial echocardiogram showed dramatic improvement in cardiac function. The left ventricular volume normalized, and the ejection fraction improved to 55%. The right ventricle normalized in size and function. Moderate concentric thickening of the left ventricle persisted with a maximum wall thickness of 17 mm. Delayed enhancement imaging was normal (Figure 3).

## 2. Discussion

Carnitine (3-hyrdoxy-4-N-trimethylaminobutyrate) is an amino acid compound that is essential for cellular metabolism. It facilitates transfer of cytosolic long-chain fatty acids across the mitochondrial membrane for B-oxidation, providing a substrate for the oxidative phosphorylation cascade. Primarily obtained from dietary sources such as meat and dairy, 99.5% of total body carnitine is stored intracellularly and 0.5% circulates as unbound carnitine in the plasma. Plasma carnitine is renally excreted via free filtration across the glomerulus but under normal conditions is highly conserved with 95–99% tubular resorption. Intracellular concentration and tubular resorption of carnitine are facilitated by OCTN2, a widely expressed high-affinity carnitine plasma transport protein. Dysfunction of OCTN2 leads to severe plasma and intracellular carnitine deficiency [1].

Primary carnitine deficiency is a rare autosomal recessive disorder with an incidence of one in 120,000, caused by mutations in the SLC22A5 gene, located on chromosome 5q23.3, which encodes OCTN2 [2]. The disorder most commonly presents between the ages of two and four years with cardiac or hepatic failure, which may be fatal if untreated. Some patients, however, remain asymptomatic throughout their lives. Males and females are equally affected. On histology, an affected patient's myocardium demonstrates massive lipid accumulation and fibrosis. Clinically, a dilated cardiomyopathy is observed [3].

The unique characteristic of the condition, previously reported in the paediatric literature, is the rapid reversal of cardiac dysfunction which is possible after carnitine replacement. Wang et al. illustrate this characteristic in a series of six children with the condition with severe left ventricular dysfunction. After one month of carnitine treatment, left ventricular systolic function normalized in all six patients, and after six months of therapy, the left ventricular volume also normalized [3]. To our knowledge, our case is only the second publication that describes this remarkable clinical response in an adult [4].

Findings on cardiac imaging are nonspecific. Echocardiography typically demonstrates left ventricular thickening, left ventricular dilation, depressed left ventricular systolic function, and thickening of mitral valve papillary muscles and trabeculae [5].

Our case is only the second that describes MRI findings in an adult patient. The first published case described a 23-year-old woman with a 10-year history of intermittent and prolonged nonadherence with carnitine therapy. This patient's MRI demonstrated patchy late gadolinium enhancement (LGE), implying myocardial fibrosis. The absence of any LGE in our patient is an important feature that differentiates the two cases, which we believe provides unique insight into the natural history of the condition. We suggest that sustained periods of carnitine nonadherence in the first patient resulted in myocardial necrosis due to prolonged alterations in cellular metabolism. Our patient, who had a comparatively brief interruption in her therapy, did not develop this, suggesting that myocardial damage can be prevented by continuous carnitine therapy. This suggestion is supported by other cases in the literature that describe appropriately treated adult patients with up to 30 years of follow-up without left ventricular dysfunction or malignant arrhythmia [3].

The observed changes of enlarged peaked T waves and short QT interval seen in our patient are similar to those described in the paediatric cases and are characteristic of the condition. They are regarded as markers of electrical instability that predispose to ventricular arrhythmia. A majority of patients' ECGs normalize after sustained normalization of plasma carnitine concentration; however, patients remain at increased risk of sudden cardiac death [6].

## References

[1] El-Hattab A. W., Scaglia F. (2015). Disorders of carnitine biosynthesis and transport. *Molecular Genetics and Metabolism*.

[2] Magoulas P. L., El-Hattab A. W. (2012). Systemic primary carnitine deficiency: an overview of clinical manifestations, diagnosis, and management. *Orphanet Journal of Rare Diseases*.

[3] Fu L., Huang M., Chen S. (2013). Primary carnitine deficiency and cardiomyopathy. *Korean Circulation Journal*.

[4] Ascunce R. R., Nayar A. C., Phoon C. K., Srichai M. B. (2013). Cardiac magnetic resonance findings in a case of carnitine deficiency. *Texas Heart Institute Journal*.

[5] Wang S. S., Rao J., Li Y. F., Zhang Z. W., Zeng G. H. (2014). Primary carnitine deficiency cardiomyopathy. *International Journal of Cardiology*.

[6] Roussel J., Labarthe F., Thireau J. (2016). Carnitine deficiency induces a short QT syndrome. *Heart Rhythm*.

